# Moisture-Resistant, High-Performance Polarizing Films via Aligned PMMA/CNT Composite Fibers: A Scalable Electrospinning Approach

**DOI:** 10.3390/molecules30102169

**Published:** 2025-05-15

**Authors:** Yanyu Gao, Xueyang Chen, Yunjie Zhang, Xue-Hui Dong, Qianqian Yu, LinGe Wang

**Affiliations:** South China Advanced Institute for Soft Matter Science and Technology, School of Emergent Soft Matter, Guangdong Provincial Key Laboratory of Functional and Intelligent Hybrid Materials and Devices, Guangdong Basic Research Center of Excellence for Energy and Information Polymer Materials, South China University of Technology, Guangzhou 510640, China; gaoyanyu0821@163.com (Y.G.); yuqianqian@scut.edu.cn (Q.Y.)

**Keywords:** polarizers, poly(methyl methacrylate), carbon nanotubes, electrospinning, composite fibers

## Abstract

Traditional iodine-based polyvinyl alcohol (PVA) polarizers encounter considerable durability challenges, especially in humid conditions, due to poor moisture resistance. This study presents an innovative organic–inorganic composite film composed of poly(methyl methacrylate) (PMMA) and carbon nanotubes (CNTs), fabricated via electrospinning, solvent vapor annealing (SVA), and uniaxial stretching. Pre-aligned PMMA/CNT composite fibers were electrospun and underwent SVA to stabilize the structure and reduce inter-fiber porosity. Further uniaxial stretching aligned the CNTs, enhancing optical anisotropy and polarization performance. The optimized parameters, 45 min of SVA and 75% stretching strain, produced composite films with a polarization degree exceeding 60%, which was combined with exceptional moisture resistance (<2% weight variation under 90% relative humidity). The integration of CNTs enhanced mechanical stability while preserving alignment during post-processing, thereby tackling the crucial challenge of scalable nanomaterial orientation. This study provides a scalable, cost-effective approach for developing durable polarizing materials with enhanced performance for optical devices in demanding environments.

## 1. Introduction

Polarizers are essential components in optical devices such as displays, sensors, and imaging systems, enabling selective light transmission based on polarization states [1,2,3,4,5]. However, their widespread application is often hindered by durability issues, especially in humid environments [6]. This limitation highlights the critical demand for developing robust polarizing materials that retain functionality under harsh operational conditions. Recent breakthroughs in materials science highlight novel organic–inorganic composites as promising solutions, offering enhanced performance and durability for next-generation polarizing films [7,8,9].

One-dimensional (1D) nanomaterials, including nanowires, nanotubes, and nanofibers, have garnered significant attention for their potential in the development of advanced polarizers, owing to their distinctive optical anisotropy and high aspect ratio [10,11,12,13,14,15]. The primary challenge in employing 1D nanomaterials for polarization applications pertains to the achievement of macroscopic alignment. Achieving optimal performance necessitates the orientation of the nanomaterials in a consistent direction, a feat that is hindered by their propensity to aggregate or align randomly during processing. A number of techniques have been developed to address the issue under discussion. These include shear-induced alignment [16,17], electromagnetic fields assistance [18,19,20,21], and mechanical rubbing [22,23]. However, despite the exploration of these methods, achieving a robust, large-scale alignment of 1D nanomaterials remains a significant hurdle for practical applications.

Poly(methyl methacrylate) (PMMA) was first discovered in the 1930s and initially applied by Otto Rohm in 1934 [24]. It is renowned for its excellent optical properties, mechanical stability, and chemical resistance. Its lightweight nature and superior transparency make it a popular alternative to glass substitutes in applications such as optical devices and coatings [25]. Its high impact strength, low density, excellent transparency, and favorable processability make it an attractive material. Furthermore, PMMA exhibits remarkable weatherability, maintaining its structural and functional integrity under various environmental conditions [26,27]. PMMA’s resistance to UV radiation, chemical durability, and mechanical robustness have established it as a key material for lenses, microscopes, lasers, and other optical applications. These attributes have solidified PMMA’s position as a versatile material in diverse industrial and research domains. In recent years, PMMA fibers have attracted considerable attention due to their distinctive properties and augmented functionality when processed into fibrous forms [28,29].

Electrospinning is a method of creating nanofibers that is known for its cost effectiveness and ability to produce fibers with tunable structures over large areas [30,31,32]. Aligned fibers produced via electrospinning can serve as carriers to guide the preferential alignment of anisotropic 1D nanomaterials along the fiber axis [33,34,35,36]. This process can be used to achieve macroscopic orientation and enhance optical properties such as polarization effects. Consequently, the integration of 1D nanomaterials into a polymer matrix, as exemplified by electrospun nanofibers, is a promising solution. The electrospinning process not only facilitates the alignment of these materials but also provides a straightforward method to scale up production, rendering it suitable for industrial applications [37,38]. However, electrospun fibers are frequently opaque due to light reflection and refraction at the fiber–air interfaces, and their absence of inter-fiber interactions can result in deformation and reduced structural integrity, thus limiting their practical applications. Techniques such as solvent vapor annealing [39,40] (SVA) and uniaxial stretching can further align polymer chains and carbon nanotubes (CNTs), enhancing optical anisotropy and polarization performance in composite films. Zhou et al. [41] showed that the mechanical stretching method can effectively modulate the degree of CNTs alignment in the polymer matrix. By adjusting the stretching ratio, it is possible to optimize the alignment ratio and tessellation angle, and the method can be extended to other 1D nanostructures. Siegrist et al. [42] demonstrated that stretching promotes the ordering of nanotube bundles in the stretching direction by reducing their wavy bending.

This study addresses the critical limitation of conventional iodine-based polyvinyl alcohol (PVA) polarizers, poor moisture resistance, by introducing an innovative organic–inorganic composite fiber strategy. The approach aims to simultaneously enhance humidity resistance and optical anisotropy in polarizing films. By incorporating CNTs into PMMA fibers, we developed composite films with superior moisture stability and polarization performance. The process involves pre-aligning CNTs within PMMA fibers via electrospinning, consolidating the fibers into films through SVA, and further optimizing alignment via uniaxial stretching. The resulting anisotropic PMMA/CNT films were rigorously characterized using scanning electron microscopy (SEM), polarized optical microscopy, and UV–Vis spectroscopy to evaluate the influence of CNT concentration, annealing time, and stretching on optical properties. This work provides a comprehensive framework for designing advanced polarizing materials with tailored functionalities through precise control of processing and post-treatment parameters.

## 2. Results and Discussion

### 2.1. Characterization of PMMA Fiber

**Figure 1:**
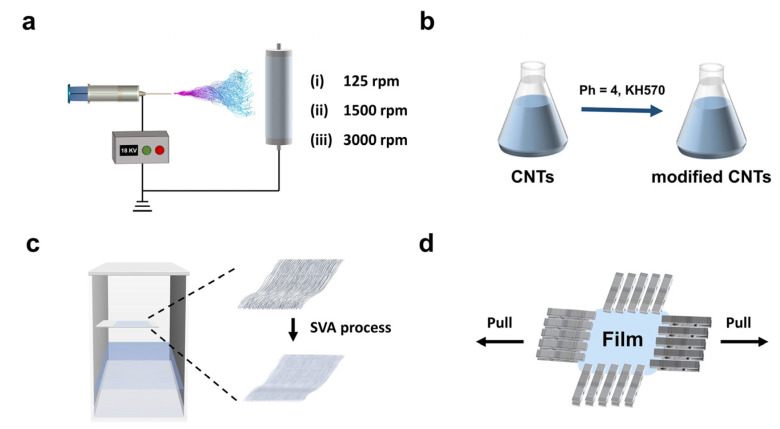
(**a**) The process and device of electrospinning aligned fibers; (**b**) preparation of silane-modified CNTs; (**c**) SVA process of fibers; and (**d**) schematic diagram of the uniaxial stretching device of SVA fibers.

**Figure 2:**
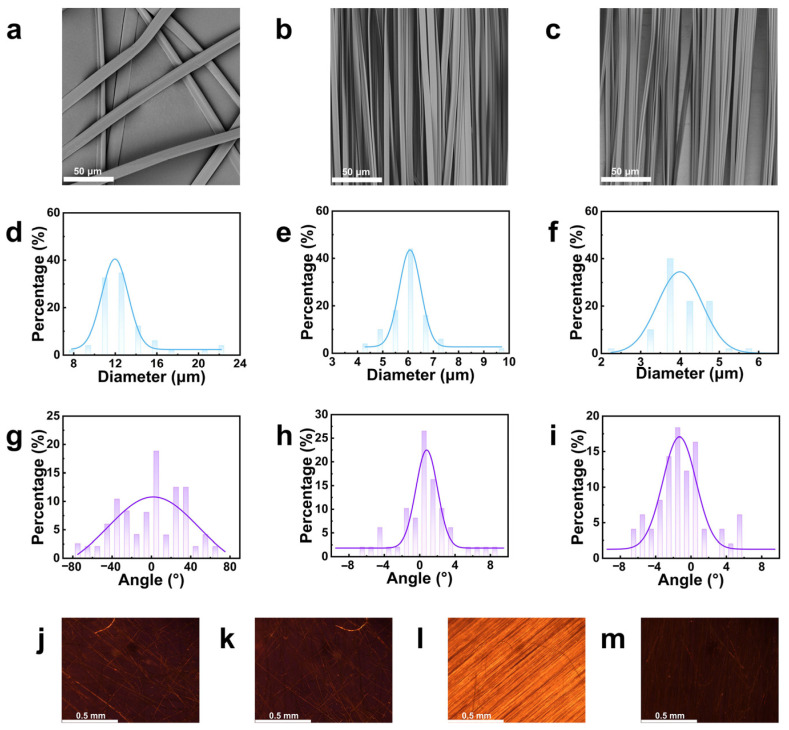
SEM image of PMMA electrospun fibers with different collector rotation speed and histograms of diameter and orientation angle distribution: (**a**) 125 r min^−1^; (**b**) 1500 r min^−1^; (**c**) 3000 r min^−1^. (**d**) Diameter and (**g**) orientation angle distribution histograms of PMMA electrospun fibers when collector rotation speed was 125 r min^−1^; (**e**) histograms of diameter and (**h**) orientation angle distribution histograms of PMMA electrospun fibers when collector rotation speed was 1500 r min^−1^. Histograms of (**f**) diameter and (**i**) orientation angle distribution histograms of PMMA electrospun fibers when collector rotation speed was 3000 r min^−1^; Polarizing microscope images of PMMA fiber: (**j**) the polarization direction of random PMMA fibers with 125 r min^−1^ was parallel to the polarization direction of incident light; (**k**) the polarization direction of random PMMA fibers with 125 r min^−1^ was perpendicular to the polarization direction of the incident light; (**l**) the polarization direction of oriented PMMA fibers with 1500 r min^−1^ was parallel to the polarization direction of incident light; (**m**) the polarization direction of oriented PMMA fibers with 1500 r min^−1^ was perpendicular to the polarization direction of incident light.

PMMA fibers were prepared by electrospinning, as shown in Figure 1. The effect of collector rotation speed on fiber diameter and orientation was investigated by varying the collector speed. Scanning electron microscopy (SEM) images in Figure 2a–c show that at a rotation speed of 125 r min^−1^, the fibers are randomly distributed with no alignment. At 1500 r min^−1^, the fibers align predominantly along the rotation direction. Increasing the collector speed further from 1500 r min^−1^ to 3000 r min^−1^ did not result in significant changes in fiber orientation. The average fiber diameters, measured using ImageJ 1.54g software, were found to be 13.07 ± 3.37 μm, 6.02 ± 0.86 μm, and 4.06 ± 0.62 μm, respectively (Figure 2d–f). The percentage of fibers with an alignment angle within 5° of the maximum fiber orientations was calculated, as shown in Figure 2g–i. The values were 20.00%, 87.76%, and 91.84%, respectively, indicating that fiber alignment improves with increasing collector rotation speed. This is likely due to the enhanced jetting force generated by the rotating drum.

The optical anisotropy of PMMA fibers with different orientations was examined using a polarizing microscope. When natural light passes through two perpendicular polarizers, the field of view appears dark as all light is blocked. However, placing an optically anisotropic material between the polarizers causes the light to deviate, resulting in changes in brightness. As shown in Figure 2j–m, the fibers obtained at 125 r min^−1^ show no change in brightness, indicating a random fiber distribution. At 1500 r min^−1^, the fiber membrane exhibits clear brightness variations as the turntable rotates. When the fiber orientation angle is 45° relative to the perpendicular direction, clear alignment is observed, and the field gradually darkens as the angle shifts. This suggests that only oriented PMMA fibers exhibit optical anisotropy, likely due to the arrangement of the fibers in the PMMA matrix, which leads to different refractive indices along two orthogonal directions.

Thus, PMMA fibers with controlled orientation were successfully prepared using a collector rotation speed of 1500 r min^−1^.

### 2.2. The Effect of CNTs Concentration on the Morphology and Properties of PMMA/CNTs Composite Fibers

**Figure 3:**
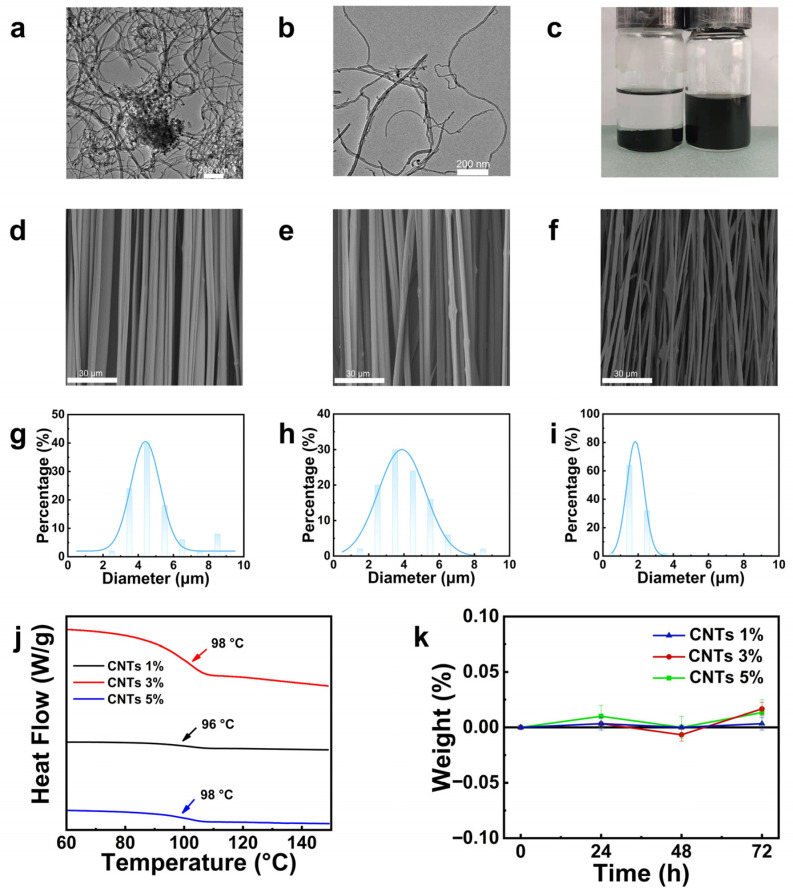
(**a**) TEM image of unmodified CNTs; (**b**) TEM image of modified CNTs; (**c**) digital photo of CNTs dispersion before and after modification for 24 h (left: unmodified; right: modified). SEM image and histograms of diameter of PMMA electrospun fibers with different CNTs contents: (**d**) SEM image and (**g**) histograms of diameter of PMMA electrospun fibers with CNTs 1 wt%; (**e**) SEM image and (**h**) histograms of diameter of PMMA electrospun fibers with CNTs 3 wt%; (**f**) SEM image and (**i**) histograms of diameter of PMMA electrospun fibers with CNTs 5 wt%; (**j**) DSC heating curve of PMMA/CNTs composite film with different CNTs content; (**k**) moisture absorption weight change of PMMA/CNTs composite film with different CNTs content.

The surface of CNTs was modified with a silane coupling agent to improve their dispersibility. The characteristic peaks at 1724 cm^−1^, corresponding to the C=O bond, and at 1079 cm^−1^, corresponding to Si-O-CH_3_, confirm the successful modification of the CNTs surface [43]. TEM images of the modified CNTs (Figure 3a,b) show reduced entanglement between the CNTs, indicating better dispersion. After ultrasonic treatment and standing for 24 h in an ethanol solution, the silane-modified CNTs remained uniformly dispersed, while unmodified CNTs precipitated (Figure 3c), demonstrating the improvement in dispersibility due to the silane modification.

PMMA/CNTs composite fibers with CNTs concentrations of 1 wt%, 3 wt%, and 5 wt% were prepared and characterized by SEM (Figure 3d–f). The average fibers diameters, calculated using ImageJ software, were 4.82 ± 1.44 μm, 4.08 ± 1.33 μm, and 1.87 ± 0.51 μm, respectively (Figure 3g–i). The fiber diameter decreased as the CNTs content increased, which can be attributed to the enhanced electrical conductivity of CNTs. As the concentration of CNTs in the solution increases, the electrostatic charge on the fibers surface grows, amplifying the electrostatic force during electrospinning and resulting in greater fibers elongation. However, as the concentration of CNTs in the polymer solution is increased, a corresponding aggregation is observed. This aggregation leads to a less uniform and smooth fiber morphology and an increased fibers gap width (see Figure 3f). As demonstrated in Figure S2, the CNTs 1 wt% composite fibers exhibit the highest polarization degree of over 60%. However, an increase in CNTs content has been observed to result in a decrease in polarization degree. This phenomenon may be attributed to the enhanced agglomeration of CNTs, which occurs as their content rises. This leads to an increase in CNTs disorder within the fibers. Consequently, there is a decrease in fiber morphology uniformity and a reduction in the smoothing effect. Consequently, the polarization degree is reduced.

The DSC curves of the PMMA/CNTs composite fibers with varying CNTs contents (Figure 3j) show that the incorporation of CNTs has minimal effect on the glass transition temperature (*T*_g_), suggesting that there is no significant interaction between CNTs and PMMA, and the composite behaves as a physical blend. Mechanical properties of the PMMA/CNTs composite fibers with different CNTs concentrations are summarized in Table S1. As the CNTs content increased, the tensile strength decreased to 9.4 MPa, 6.3 MPa, and 4.7 MPa, and the elastic modulus decreased to 618.2 MPa, 540.6 MPa, and 524.4 MPa, respectively. Firstly, the introduction of carbon nanotubes plays a role in plasticizing to a certain extent, and at the same time, the fibers diameter decreases due to the nanotubes adhering to the fibers surface, resulting in the fibers gap increasing with increasing carbon nanotube concentration (Figure 3d–f), which weakens the inter-fibers interactions and thus reduces the mechanical properties of the film.

To address the moisture sensitivity and associated degradation of iodine-based PVA polarizers, the moisture stability of the PMMA/CNTs composite fibers was evaluated (Figure 3k). The results showed minimal weight change over 72 h at 40 °C and 90% relative humidity, indicating that the composite fibers exhibit good moisture resistance.

### 2.3. Effect of SVA on the Morphology and Properties of PMMA/CNTs Composite Fibers

To investigate the impact of pore structure on fibers performance, PMMA/CNTs composite fibers with 1 wt% CNTs concentration and optimal orientation were subjected to SVA. This process consolidates the fibers into a film structure, reducing pore spaces between them and minimizing light energy loss. SVA generates free volume for molecular chain movement, with the annealing time influencing the extent of this movement. The primary focus of this study is the effect of annealing time on the morphology and properties of PMMA/CNTs composite optoelectronic films.

**Figure 4:**
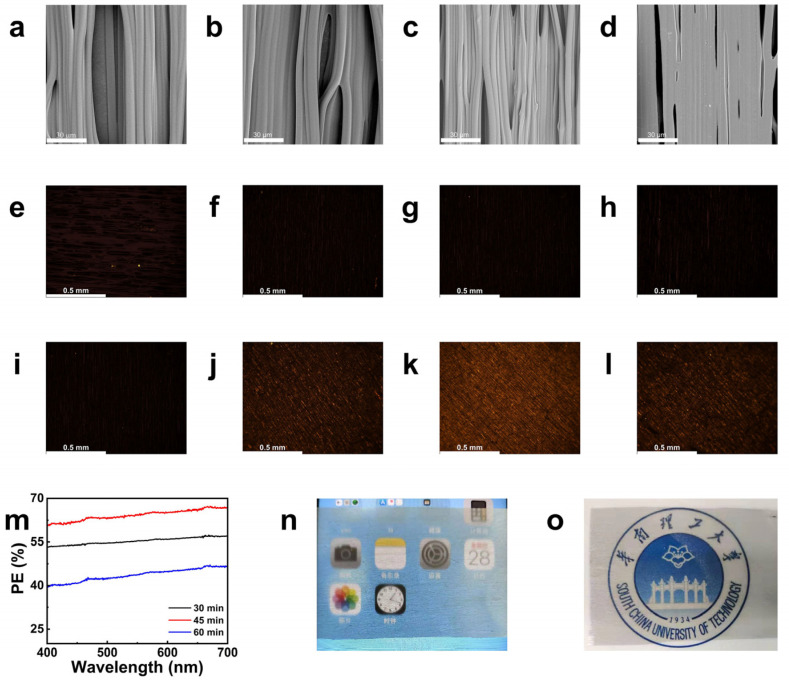
(**a**,**e**,**i**) SEM image and polarizing microscope of oriented PMMA fibers with 1500 r min^−1^ after annealing for 45 min; (**b**,**f,j**) SEM image and polarizing microscope of PMMA/CNTs composite film after solvent vapor annealing for 30 min; (**c**,**g,k**) SEM image and polarizing microscope of PMMA/CNTs composite film after solvent vapor annealing for 45 min; (**d,h,l**) SEM image and polarizing microscope of PMMA/CNTs composite film after solvent vapor annealing for 60 min; (**m**) polarization degree of PMMA/CNTs composite film after different solvent vapor annealing times; Digital photo of PMMA/CNTs composite film after solvent vapor annealing for (**n**) 30 min and (**o**) 45 min.

SVA was performed for 30, 45, and 60 min on 1 wt% CNTs PMMA/CNTs composite fibers, and the morphological changes were analyzed using SEM (Figure 4b–d). As the annealing time increased, the bonding between fibers became more pronounced, and after 60 min, the fiber morphology nearly vanished. The polarization degree was measured using UV–Vis spectroscopy (Figure 4m) and calculated using Formula (1), where $T_{∥}$ is the transmittance for films with parallel orientation and $T_{⊥}$ is the transmittance for films with perpendicular orientation. The polarization degree increased with annealing time, reaching its maximum at 45 min before decreasing at 60 min. This trend was consistent with the observations from polarized optical microscopy (Figure 4f–h,j–l). During SVA, the rearrangement of the polymer molecular chains and the displacement of the CNTs induce interfacial shear stresses: a mismatch in the thermal expansion coefficients between the CNTs and the PMMA matrix. Residual stresses can stabilize the orientation of the CNTs by inhibiting the thermal movement of the molecular chains. Therefore, optimizing the annealing time is essentially a key process to balance the stress enhancement effect with structural damage. The high mobility of the polymer chains during the continuous erosion of the fibers by solvent vapor also affects the fibers microstructure [39]. After 30 min of SVA, inter-fiber fusion started (Figure 4b). Porous fiber scaffolds with micron-sized pores were obtained after 45 min (Figure 4c). During this process, the orientation of the fibers and the arrangement of the CNTs within the fibers were not disturbed. When the exposure time is increased to 60 min (stage 3, Figure 4d), prolonged annealing leads to a deterioration of the fiber morphology and disruption of the arrangement of the CNTs within the fibers due to molecular chain motion. These results are consistent with our previous findings where microstructural evolution was observed in annealed fiber scaffolds to produce different morphologies [39], showing the topological evolution process of annealed fibers and demonstrating the effect of SVA on the microstructure, nanostructure, and related properties of fibers.

Figure 4e,i show polarized optical micrographs of pure PMMA fibers annealed for 45 min, while Figure 4g,k show the same annealed time for PMMA/CNTs composite fibers. The results indicate that pure PMMA fibers lose their optical anisotropy after 45 min of annealing, whereas the PMMA/CNTs composite fibers retain contrasting dark and bright fields under polarized light. This suggests that CNTs help maintain the fibers’ morphology and enhance optical anisotropy.

(1) $$E=\sqrt{\frac{T_{∥}-T_{⊥}}{T_{∥}+T_{⊥}}}\times 100\%,$$

### 2.4. The Effect of Stretching Amplitude on the Morphology and Properties of PMMA/CNTs Composite Films

**Figure 5:**
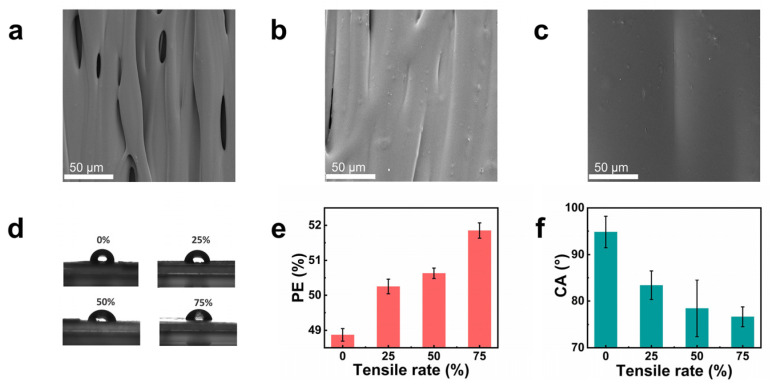
SEM images of PMMA/CNTs composite films after annealing for 45 min with different tensile rates: (**a**) 25%; (**b**) 50%; (**c**) 75%. (**d**) The photos of PMMA/CNTs composite CA films with different tensile rates. (**e**) Polarization degree of PMMA/CNTs composite films with different tensile rates. (**f**) CA of PMMA/CNTs composite films with different tensile rates.

To investigate the effect of further film orientation on polarization performance, PMMA/CNTs composite films that were annealed for 45 min were subjected to uniaxial stretching at varying extents. The morphological changes at stretching strains of 25%, 50%, and 75% were characterized using SEM (Figure 5a–c). The SEM images show that as the stretching strain increased, the fiber morphology of the PMMA/CNTs composite films gradually disappeared, and the pores in the composite films became smaller. This could be due to the uniaxial stretching process being conducted at 120 °C, above the *T*_g_, where the stretching rate remained constant. As the strain increased, the stretching time also increased, and with the temperature staying above *T*_g_, the PMMA molecular chains underwent reorientation and rearrangement, essentially performing a thermal annealing process. This led to an increase in the adhesion of PMMA/CNTs composite fibers film and the loss of fiber morphology. Additionally, contact angle (CA) wetting tests were performed on the composite films at different stretching strains (Figure 5d). The results showed that as the stretching strain increased, the contact angle gradually decreased, indicating that the surface morphology of the PMMA/CNT composite films was altered by the stretching. Firstly, the oriented fiber gap decreases with increasing elongation in uniaxial stretching, which is attributed to its macroscopic orientation along the stretching direction [44]. Furthermore, an increase in fibers diameter results in an augmentation of the contact surface between the water droplet and the film surface, consequently leading to a reduction in the contact angle [45].

Using a UV–Vis spectrometer, the transmittance of two fiber membranes oriented parallel and perpendicular to each other was measured, and the polarization degree was calculated using Formula (1) for different stretching strains, as shown in Figure 5e. The data show that as the stretching strain increased, the polarization degree also increased, suggesting that uniaxial stretching can further orient the CNTs, thereby enhancing the optical anisotropy of the films.

## 3. Materials and Methods

### 3.1. Materials

PMMA (*M*_n_~80,000) was obtained from Arkema (Shanghai, China). Trichloromethane (TCM), hydrochloric acid (HCl), hexane, and ethanol were obtained from Guangzhou Chemical Reagents Co., Ltd. (Guangzhou, China) Silane coupling agent (KH570) was purchased from Aladdin (Singapore). Carbon nanotubes (CNTs, 0.5–2 μm) were obtained from Suzhou Carbon Rich Technology Co., Ltd. (Suzhou, China). *N*,*N*-dimethylformamide (DMF) and ethyl acetate (EA) were purchased from Shanghai Titan Technology Co., Ltd. (Shanghai, China).

### 3.2. Preparation of Light-Functional Fibers Film

#### 3.2.1. Preparation of PMMA Fibers

To prepare a 20 wt% PMMA solution, PMMA powder was dissolved in TCM (2 g of PMMA, 8 g of TCM). A handmade uniaxial electrospinning machine was applied to manufacture the fibers [46]. As shown in Figure 1a, the electrospinning parameters were set as follows: a spinning voltage of 18 kV and a working rate of 2 mL h^−1^. A drum was used as the collector with a diameter of 10 cm. The drum speed was set at 125 rpm, 1500 rpm, and 3000 rpm, with a deviation of no more than 5%. The collection distance was set to 20 cm, and the average ambient temperature was 26 °C. Subsequent to electrospinning, the fibre membrane was subjected to vacuum oven drying at 30 °C for a period of 24 h, with the objective of eliminating residual solvent.

#### 3.2.2. Preparation of PMMA/CNTs Composite Fibers

Firstly, modified CNTs were prepared. The CNTs were dispersed in a 1:1 ethanol/water solution using an ultrasonic cell disruptor. The pH of the solution was adjusted to 4 with diluted HCl solution. KH570 was then added dropwise during the ultrasonic stirring process, and the reaction was allowed to proceed at 30 °C for 12 h. Afterward, the mixture was centrifuged, washed, and dried at 60 °C for 24 h to obtain the silane-modified CNTs.

Next, an appropriate amount of PMMA and CNTs was mixed and dispersed in TCM solvent using ultrasonic treatment for 10 min. PMMA/CNTs solutions with CNT concentrations of 1 wt%, 3 wt%, and 5 wt% were prepared. Electrospinning was performed under the same parameters as described in Section 3.2.1.

### 3.3. Solvent Vapor Annealing (SVA) of Fibers

The fibers, along with aluminum foil, were cut into strips of 80 × 50 mm in size and placed into a custom-made annealing device for solvent vapor annealing experiments (Figure 1c). Ethyl acetate was selected as the solvent, and the SVA was conducted at a temperature of 40 °C for durations of 30, 45, and 60 min. After the SVA process, the films were allowed to cool and dry at 30 °C.

### 3.4. Uniaxial Stretching of SVA Fibers

The SVA fibers film were peeled off from the aluminum foil and cut into rectangular strips of 80 × 50 mm. Uniaxial stretching was then performed using a biaxial film stretching machine (Figure 1d) (KARO IV model, Brückner, Siegsdorf, Germany). The specific experimental procedure is as follows: The strips were first preheated for 60 s in the stretching heating chamber of the biaxial film stretcher. Subsequently, uniaxial stretching was conducted at a stretching speed of 8 mm s^−1^ and a temperature of 125 °C, with different stretching amplitudes of 25%, 50%, and 75%.

## 4. Conclusions

This study presents an exacting method for fabricating high-performance PMMA/CNT composite polarizing films using electrospinning, SVA, and uniaxial stretching. The integration of silane-modified CNTs into the PMMA matrix markedly improved moisture resistance, exhibiting minimal weight alteration (<2%) under extreme humidity conditions (90% RH, 72 h). SVA successfully consolidated the electrospun fibers, reducing pore size and enhancing structural integrity, while uniaxial stretching induced macroscopic alignment of CNTs, achieving a polarization degree exceeding 60%. Systematic optimization revealed that 45 min of SVA and 75% stretching strain maximized optical anisotropy without compromising mechanical stability. These findings highlight the dual role of CNTs for enhancing both durability and polarization efficiency, overcoming the limitations of conventional iodine-based PVA polarizers. The scalable electrospinning process, along with appropriate post-treatments, establishes this approach as a viable pathway for manufacturing advanced optical materials. The developed composite films show promise for applications in flexible displays, humidity-resistant sensors, and energy-efficient optical devices, facilitating the transition from laboratory discovery to industrial implementation. Future endeavors will concentrate on customizing CNT–polymer interactions for multifunctional optical devices and on large-scale manufacturing methods.

## Data Availability

The original contributions presented in this study are included in the article and Supplementary Materials.

## Supplementary Materials

The following supporting information can be downloaded at: https://www.mdpi.com/article/10.3390/molecules30102169/s1, Section S1: Characteristic; Figure S1: The FT-IR spectrum of modified CNTs; Figure S2: Polarization of composite fibers with different CNTs content; Table S1: The solubility of polyimides.
