# Supplementary material for: Moisture-Resistant, High-Performance Polarizing Films via Aligned PMMA/CNT Composite Fibers: A Scalable Electrospinning Approach
Source: Molecules. 2025 May 15;30(10):2169. doi: 10.3390/molecules30102169 (PMC12113673; doi:10.3390/molecules30102169)
Supplement: Supplementary file 1 [file molecules-30-02169-s001.zip › molecules-3580236-supplementary.pdf]

SUPPORTING INFORMATION

Moisture-Resistant, High-Performance Polarizing Films via Aligned PMMA/CNT Composite Fibers: A Scalable Electrospinning Approach

Yanyu Gao [†], Xueyang Chen [†], Yunjie Zhang, Xue-Hui Dong, Qianqian Yu and LinGe Wang *

South China Advanced Institute for Soft Matter Science and Technology, School of Emergent Soft Matter, Guangdong Provincial Key Laboratory of Functional and Intelligent Hybrid Materials and Devices, Guangdong Basic Research Center of Excellence for Energy and Information Polymer Materials, South China University of Technology, Guangzhou 510640, China; gaoyanyu0821@163.com (Y.G.); yuqianqian@scut.edu.cn (Q.Y.)

* Correspondence: lingewang@scut.edu.cn

† These authors contributed equally to this work.

Table of contents

S1. Experimental Section 3

Characteristic 3

S2. Supplemental Figures 4

S3. Supplemental Table 6

S1. Experimental Section

Characteristic

SEM was performed on a Phenom Pro G3 (Phenom, Holland). To enhance the signal quality, samples were firstly sputtered with a layer of platinum before examination. The diameter distribution of fibers was analyzed by ImageJ software. (Transmission Electron Microscopy, TEM) was performed on JEM-1400plus, with an operating voltage of 120 kV. The samples were cut into a strip with 50 × 20 mm for tensile testing on a universal testing machine (LABSANS LD22.102) in accordance with the prevailing ASTM Test Standards (ASTM C1557) The testing speed was set at 5 mm min-1. The optical anisotropy of fibers was observed using an Olympus BH2 polarizing microscope. Following the peeling of the electrospun oriented fibers from the aluminum foil, they were in a horizontal position on a glass slide, which was then placed on a 360° rotating turntable. After focusing the sample, the turntable was rotated at different angles, and the variation in brightness and darkness in the field of view was observed and photographed. The samples were subjected to a series of tests to ascertain their weight change in a constant temperature and humidity chamber (PL-150) at 40 °C and 90% RH. Samples were weighed at 24 h intervals, and three parallel samples were taken from each group to calculate the average weight change and standard deviation. The ultraviolet-visible (UV-Vis) spectrophotometer was used for testing, in accordance with the national standard GB/T 25275-2010. Air was employed as the reference medium. The wavelength range was configured to encompass 400 – 700 nm. The parallel transmittance of the samples was measured at each wavelength under parallel fiber orientation, and the vertical transmittance was measured at each wavelength under perpendicular fiber orientation.

S2. Supplemental Figures

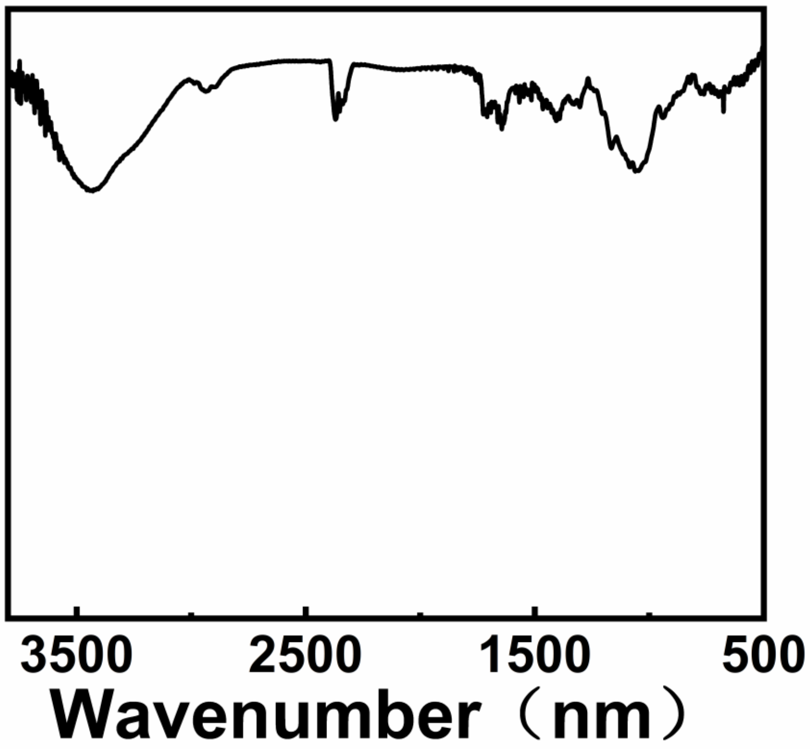

Figure S1. The FT- IR spectrum of modified CNTs.

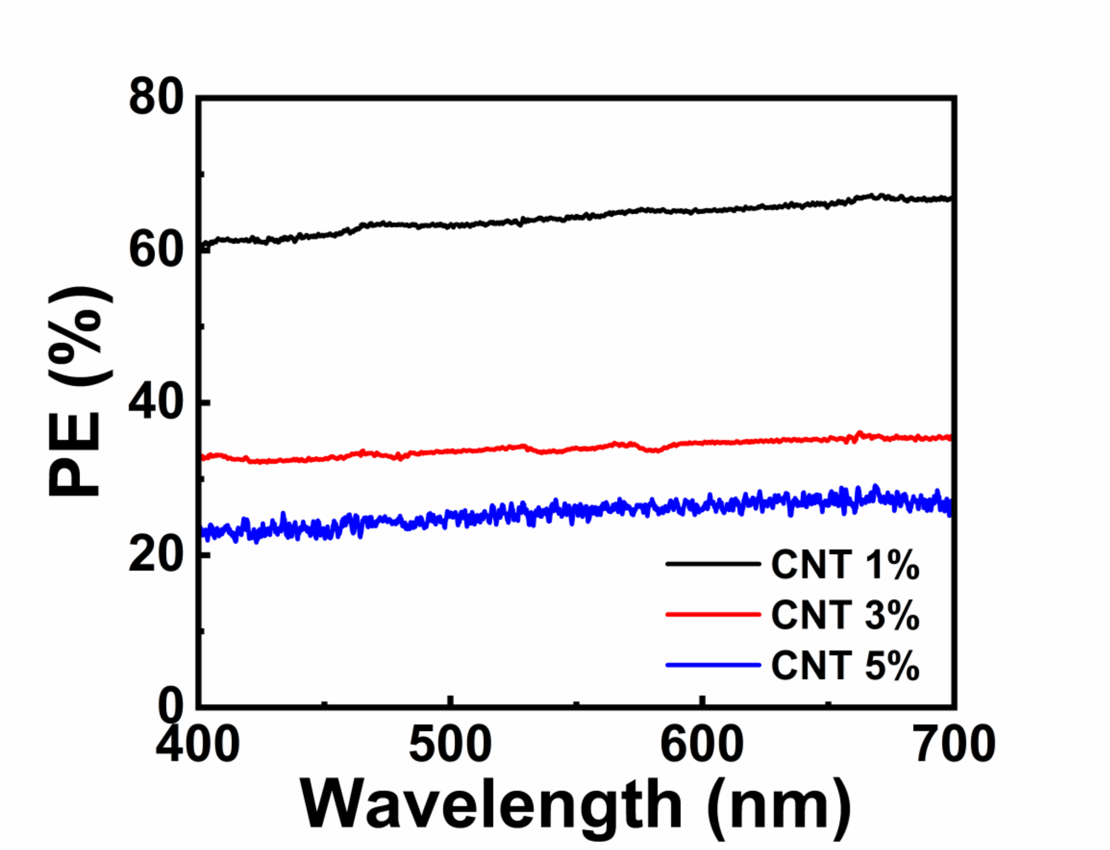

Figure S2. Polarization of composite fibers with different CNTs content.

S3. Supplemental Table

Table S1. The solubility of polyimides.

**PMMA/CNTs composite fibers**	**Concentrations of CNTs (%)**		
	**1**	**2**	**3**
Tensile Strength (MPa)	9.4	6.3	4.7
Elastic Modulus (MPa)	618.2	540.6	324.4
